# Cyclosporine-induced Erythromelalgia

**DOI:** 10.7759/cureus.3506

**Published:** 2018-10-27

**Authors:** Lorin A Bibb, Randi P Winter, Stuart S Leicht

**Affiliations:** 1 Dermatology, East Tennessee State University Quillen College of Medicine, Johnson City, USA

**Keywords:** erythromelalgia, cyclosporine, erythema, edema, vasodilation, distal extremities, secondary erythromelalgia, erythermalgia, cyclosporin, neoral

## Abstract

Erythromelalgia is a neurovascular disorder which causes pain, swelling, erythema, and warmth of the distal extremities. Primary disease is due to a genetic mutation in the *SCN9A* gene, but secondary erythromelalgia can be the consequence of a variety of underlying etiologies, including drug and toxin exposures. The disease is rare, occurring in only 1.3 out of every 100,000 in the United States, and symptoms can vary significantly in severity and presentation. Therefore, it can be difficult to recognize the disorder, identify the source, and promptly treat the condition. We report a reversible cause of erythromelalgia induced by the use of oral cyclosporine. This correlation is poorly documented in literature, with limited accounts identifying an association between erythromelalgia and cyclosporine. As drug-induced erythromelalgia represents a reversible cause of disease, physicians should obtain a detailed medication history during the diagnostic workup, specifically inquiring about the use of cyclosporine.

## Introduction

Erythromelalgia is an uncommon disease often presenting with symptoms of erythema, warmth, and painful swelling of the extremities [1]. It can occur as a primary condition or as a secondary manifestation of another underlying disorder [1-2]. Primary erythromelalgia is a genetic disorder caused by a mutation in the gene *SCN9A* coding for the α-subunit of the Nav 1.7 voltage-gated sodium channel, which is expressed in sympathetic and sensory neurons [3-4]. A gain of function mutation leads to channel hyperexcitability in the axons of pain-sensing neurons [5]. In contrast, secondary erythromelalgia has been associated with numerous connective tissue disorders, myeloproliferative diseases, polycythemia, drug reactions, and toxin exposures. Early disease detection is often difficult due to the vast etiologies and variable symptoms [6]. Therefore, a prompt, accurate diagnosis is vital for early symptom relief, patient satisfaction, and quality of life.

Primary and secondary erythromelalgia are not classified separately as unique diagnoses, but the reported prevalence of erythromelalgia in general, is estimated at 1.3/100,000 in the United States [6]. Females are more commonly afflicted with the disease, and Davis et al. reports a two to three times increased rate in females compared to males (72.6% females and 27.4% males) [6-7]. Most patients presenting are in their middle age or late adulthood [6, 8].

## Case presentation

**Figure 1:**
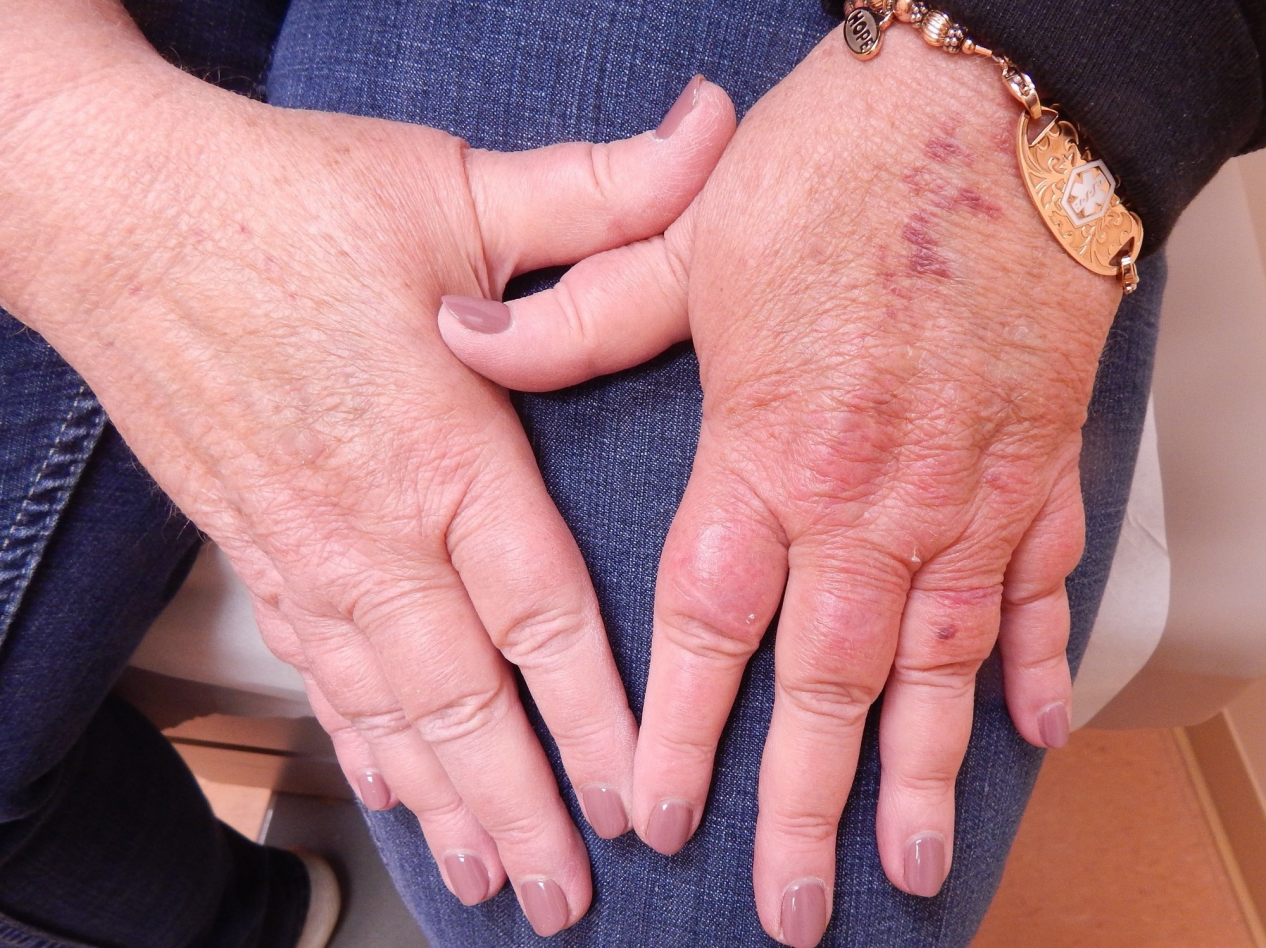
Erythema and edema after 10 weeks of cyclosporine therapy.

A 56-year-old female with a history of uncontrolled biopsy-proven bullous pemphigoid began treatment with cyclosporine 100 mg three times a day for a total daily dose of 300 mg. After five weeks of therapy, the patient complained of nervousness and shakiness, and the cyclosporine dose was decreased to 100 mg twice daily for a total daily dose of 200 mg. After an additional five weeks of cyclosporine therapy at 200 mg daily, the patient returned with complaints of the gradual onset of redness, swelling, and pain of her left hand over the past six weeks (Figure 1). On examination, erythema and violaceous swelling were present on the patient’s left hand, most predominant over the dorsal and palmar aspects of the left second and third metacarpals, and left proximal and mid phalanges. There was tenderness to palpation and pain with movement of the left hand and phalanges. The patient described the associated pain as a burning sensation. Although erythromelalgia was considered, this was believed to be a local reaction, and the cyclosporine was continued as treatment for the patient’s bullous pemphigoid.

Seven weeks later, the patient returned complaining of significantly worsened redness, swelling, and pain of the left hand with progression of involvement of the right hand. On examination, the erythema and edema involved the dorsal and palmar aspects of the left hand and phalanges, most severely affecting the left proximal phalanges. The right hand was also visibly erythematous with edema most prominently involving the right second proximal phalanx. The patient complained of worsened pain, described as “burning similar to a sunburn” that significantly worsened upon exposure to the sun or heat. The pain also worsened with passive and active movement of the hand or digits, as well as palpation of the affected areas. The patient endorsed improvement with exposure of the hands to cold temperatures, particularly describing relief of the pain while running the hands under cold water. At this time, the cyclosporine dose was decreased to 100 mg once daily with a diagnosis of erythromelalgia secondary to cyclosporine. It was hoped that a lower dose of cyclosporine could be used to treat the bullous pemphigoid, with resolution of the symptoms of erythromelalgia.

Six weeks later, the patient returned for a follow-up to evaluate the erythromelalgia. The erythema and edema had significantly improved, with only minor swelling of the second and third phalanges of the left hand and the second phalanx of the right hand. The patient's symptoms improved following the transition to low-dose cyclosporine therapy (100 mg once daily). At an additional follow-up eight weeks later, the patient’s symptoms of erythromelalgia had completely resolved.

## Discussion

Although the true pathophysiology of erythromelalgia is complicated and disputed, it is suspected that abnormalities in nerve fibers and blood flow underlie the symptoms [6]. The small nerve fibers affected are involved in sensing pain (Aδ afferent fibers) and the sympathetic nervous system (C efferent fibers) [5-6]. In a retrospective study of 52 patients with erythromelalgia examined from 2010 to 2015, Mantyh et al. found that most of these patients had epidermal nerve fiber densities within normal limits on biopsy but presented with functional abnormalities of the small nerve fibers within the epidermis. The functional abnormalities included abnormal sweat test results in 60% of the studied population, abnormal pain thresholds in 42%, and abnormal blood pressure or heart rate in 38%. Such findings can be used to distinguish erythromelalgia from similarly presenting disorders with painful distal neuropathy, including diabetes, HIV, or Fabry disease, in which patients are found to have decreased epidermal nerve fiber density [5]. In erythromelalgia, abnormalities of the small nerve fibers cause an aching, burning, and stabbing pain thought to be the result of increased perfusion through arteriovenous shunts which bypass tissue capillaries and cause ischemia [6, 9-11]. Increased flow though arteriovenous shunts ultimately decreases the oxygenated blood delivered to the capillaries of the distal extremities and causes tissue ischemia and pain [5, 6, 9-11]. Another theory suggests that vasodilatory medications can cause the pain and inflammation experienced in secondary erythromelalgia [6, 12]. Overall, it is likely an intricate interaction involving vascular dysfunction and resultant neuronal dysregulation which most likely underlies the true pathogenesis of the disease [6, 11, 13].

The diagnosis of erythromelalgia involves a thorough physical exam targeted at identifying the most common disease presentations [14]. Patients may present with episodes of burning (96%), warmth (93%), pain (87%), erythema (83%), swelling (65%), and/or numbness (54%) which typically affect the lower extremities symmetrically and bilaterally [2, 14]. The patient subjectively describes a sensation of heat affecting the area, and the erythematous extremity feels warm to palpation. The pain is usually moderate to severe and can present as sensations of burning, throbbing, pruritis, or electric shock [6]. Attacks may be intermittent, lasting approximately two to three hours with the affected area returning to normal or becoming cyanotic between episodes [6, 14]. Symptoms are often exacerbated by heat and relieved with exposure to cold [14].

At present, there is no definitive treatment for erythromelalgia, and therefore, therapy should be aimed at managing symptoms and avoiding inciting factors [1, 6]. Reported triggers include heat, pressure, extended dependent positioning of the affected limb (i.e. standing), exercise, emotions, stress, alcohol, spicy food, exposure to chronic vibration, and vasodilatory medications [15-16]. Symptoms may be episodic, with attacks lasting minutes to days, and can often be relieved by elevation, submersion of the affected limb in cold water or ice, smoking cessation, properly fitting footwear, and avoidance of vasodilatory medications [6]. The prognosis is variable depending upon the underlying cause (Table 1), and Davis et al. found that after a mean follow-up of 8.7 years, 31.9% of patients had worsened, 30.9% had improved, 10.6% were in remission, and 26.6% had no change [9]. Currently, therapeutic options include non-steroidal anti-inflammatory drugs (NSAIDs), aspirin, opioids, gabapentin, lidocaine patches, benzodiazepines, and nerve blocks [1, 17].

**Table 1:** Reported causes of secondary erythromelalgia. [2, 6, 14, 18]

Reported causes of secondary erythromelalgia
Autoimmune disorders (idiopathic thrombocytopenia, Lupus erythematosus, rheumatoid arthritis, Sjögren's syndrome)
Blood disorders (cryoglobulinemia, pernicious anemia, spherocytic anemia)
Connective tissue disorders (mixed connective tissue disease)
Drug reactions (bromocriptine, calcium channel blockers, cyclosporine, iodinated contrast agents, norephedrine, topical isopropranolol, pergolide, rosuvastatin)
Genetic diseases (Fabry disease)
Hypercholesterolemia
Hypertension
Infections
Leukemia
Leukocytoclastic vasculitis
Lichen sclerosus
Myeloproliferative disorders (polycythemia vera, essential thrombocytopenia)
Neuropathies (multiple sclerosis, neurofibromatosis, reflex sympathetic dystrophy, familial dysautonomia, small fiber neuropathies)
Paraneoplastic syndromes (astrocytoma, breast cancer, colorectal cancer, malignant thymoma, subcutaneous panniculitis-like T-cell lymphoma)
Systemic mastocytosis
Thromboembolism
Toxins (arsenic, mercury, metals, mushrooms)
Trauma, burns
Vaccines (influenza, hepatitis)
Venous insufficiency

Although a variety of drugs and toxins have been previously linked to the development of secondary erythromelalgia, very few prior case reports have documented an association with cyclosporine. In 2003, Thami and Bhalla reported a similar case of erythromelalgia presenting after four weeks of a patient taking 75 mg of cyclosporine twice daily. During this time, the patient developed erythema, edema, and tenderness of the fingers and toes. After discontinuation of the cyclosporine, the symptoms temporarily improved, but recurred when cyclosporine was restarted [15]. Based on these previous findings and the reported case above, secondary erythromelalgia can be alleviated when a causative medication is discontinued or decreased in dosage. Therefore, if cyclosporine-induced erythromelalgia is suspected, alternative pharmacotherapy options or a trial of low-dose therapy should be considered.

## Conclusions

The case presented supports previous findings suggesting that cyclosporine can cause secondary erythromelalgia, presenting with symptoms that include distal extremity warmth, redness, and pain. Based on this reported case, secondary erythromelalgia can be alleviated when the causative medication is discontinued or decreased in dosage. Because cyclosporine-induced erythromelalgia poses a reversible cause of disease, it is important for all physicians to be aware of the symptoms and potential causative medications.
